# Combating Spring Frost With Ethylene

**DOI:** 10.3389/fpls.2019.01408

**Published:** 2019-10-30

**Authors:** Jianyang Liu, Sherif M. Sherif

**Affiliations:** Alson H. Smith Jr. Agricultural Research and Extension Center, School of Plant and Environmental Sciences, Virginia Tech, Winchester, VA, United States

**Keywords:** spring frost, temperate fruits, deciduous woody perennials, bud dormancy, hormone signalling, ethylene

## Abstract

The sustainable fruit production in temperate and boreal regions is often imperiled by spring frosts. The risk of frost damage and the resulting economic losses have been increasing in the recent years as a result of the global climate change. Among the many approaches in mitigating frost damages, an ethylene-based compound, ethephon has proven to be effective in delaying bloom time in many fruit species and, thereby, avoid frost damage. However, effective concentrations of ethephon are often associated with harmful effects on fruit trees, which largely limit its use. Relatively, limited research attention has been given to understand the mechanisms underlying this ethylene-mediated bloom delay, thus hindering the progress in exploring its potential in frost protection. Recent advances in omics and bioinformatics have facilitated the identification of critical molecular and biochemical pathways that govern the progression of bud dormancy in deciduous woody perennials. In this review, we summarized our current understanding of the function of ethylene and its interaction with other networks in modulating dormancy and blooming in temperate fruit trees. Some possible mechanisms are also proposed that might potentially guide future studies attempting to decipher the dormancy regulation or searching for methods to alleviate frost damages.

## Introduction

Fruit production in temperate and boreal regions is often threatened by spring frosts. Deciduous trees lose their hardiness to cold temperature after bud break in response to rising temperatures in the spring, and frost damage may occur if the temperature drops below or near freezing again. Trees can suffer from two types of frosts: advection and radiation, with the former resulting from large cold air mass intruding an area, and the latter due to the rapid heat loss to the atmosphere, which typically occurs on calm and clear nights. In the context of global climate change, the risks of frost damage and the resultant economic losses on the global scale have been increasing at a steady pace (Augspurger, 2013). In this regard, it becomes increasingly imperative to develop technically sound and economically feasible strategies that can effectively alleviate frost damage in today’s fruit industry.

The existing methods in protecting against frost can be classified into active and passive types. Active approaches are *ad hoc* and can exhibit immediate and direct effects. Such methods usually involve the use of wind machines and helicopters to downdraft the warmer air aloft in the inversion layer 10–50 m above ground, surface irrigation by sprinklers to take advantage of the fusion heat in the water, and heaters (e.g. solid fuel, propane) to prevent frost formation. These approaches mitigate, instead of avoiding frost damage, and their efficacy is highly dependent on external factors such as frost types, wind speed, dew point, wet-bulb temperature, etc. (Tsipouridis et al., 2006; Unterberger et al., 2018). In addition, active methods are generally labor-intensive, cost-ineffective, and environmentally unsustainable. In contrast, passive methods are pre-emptive and have relatively long-lasting effects. These include breeding and selection of cold-hardy and/or late-bloom varieties, selection of proper plantation site (e.g. to avoid frost pockets), and treatment with plant growth regulators (PGR) to increase freezing tolerance (Durner, 1995; Nzokou and Paligwende, 2008), to extend the duration of bud dormancy (Durner and Gianfagna, 1991; Seeley et al., 1992), or to delay flowering time (Moghadam and Mokhtarian, 2006; Grijalva-Contreras et al., 2011). In particular, the application of PGR holds great potential in frost protection due to their high efficiency, low cost, and ease of implementation.

Endodormancy of buds is an adaptative mechanism that temperate species have evolved to survive the adverse conditions during wintertime. During endodormancy, plant regrowth is repressed by intrinsic signals and remain unresponsive to environmental cues (Rohde and Bhalerao, 2007). In this review, endodormancy will be used interchangeably with dormancy, unless specified otherwise. After entering dormancy, plants track the number of chilling hours (0–7.2 °C) and exit dormancy only when a certain number of chilling hours, also known as chilling requirement (CR), is satisfied. Chilling requirements are tightly controlled by genetics and are highly variable across species and varieties. Upon completion of dormancy, resumption of plant growth is inhibited by unfavorable environmental conditions, rather than internal cues, and this period is termed as ecodormancy (Lang, 1987). To reach full bloom stage, deciduous fruit species need to satisfy heat requirements (HRs), a period of warm temperature, which is also a genotype-dependent trait (Fan et al., 2010). Bud dormancy and regrowth cycle in deciduous woody perennials are genetically programmed, highly regulated, as well as subject to the influence of many internal and external factors.

Recent studies have also indicated that global climate change exerts profound impacts on the phenology of dormancy and flowering and the increasing risk of spring frost. Alterations in plant phenology, such as shortened dormancy and early budburst and flowering, have been observed in many woody perennials in the Northern Hemisphere (Augspurger, 2013; Ma et al., 2019). For example, apple blooming in Europe has advanced for a total of 6–9 days over the last 30 years (Vitasse et al., 2011; Hoffmann and Rath, 2013; Vitasse et al., 2018), and this trend is projected to continue for decades ahead (Unterberger et al., 2018). Combined with other consequences of the climate change such as the number of frost days during the growing season (Liu et al., 2018) and the frequency of the warm spells in the late winter or early spring (Ma et al., 2019), all these factors contribute to increasing the odds of frost damage.

## Ethylene Control of Bud Dormancy

Ethylene is a gaseous plant hormone that plays important roles in a plethora of physiological aspects, especially in responses to environmental stresses and biotic attacks (Bleecker and Kende, 2000). Ethylene is derived from methionine, which undergoes a series of conversions catalyzed sequentially by S-adenosyl methionine (SAM) synthetase, aminocyclopropane-1-carboxylic acid (ACC) synthase, and ACC oxidase (Wang et al., 2002). The conversion of SAM to ACC is a rate-limiting step, and ACC has been recognized to have similar functions as ethylene in mediating plant development and defense responses (Nascimento et al., 2018). In the ethylene signaling pathway, the endoplasmic reticulum (ER)-located ethylene receptors (ETR), upon activation by ethylene, releases Ethylene Insensitive-2 (EIN2), a positive regulator of ethylene signaling, from the repression by the protein kinase Constitutive Triple Response 1 (CTR1). Activated EIN2 migrates to nucleus to activate the transcription factors *ethylene insensitive like (EIN3/EILs)*, which in turn induces the expression of a large multigene family of *ethylene response factors* (*ERFs*) (Merchante et al., 2013). Both ethylene biosynthesis and signaling have been found to mediate in the regulation of dormancy, as application of ethylene antagonist 2, 5-norbornadiene (NBD) accelerates dormancy break in potato micro-tuber, (Suttle, 1998), and mutation with impaired ethylene reception prevents the initiation of dormancy in chrysanthemum (*Chrysanthemum morifolium*), even after treatment with high dosage of ethephon (Sumitomo et al., 2008). The finding that many genes associated with ethylene biosynthesis and signaling (e.g. *ETR2*, *EIN3*, *EIN4*, and *ERFs*) in poplar are upregulated in dormancy-inducing conditions (Ruttink et al., 2007) also reinforces the notion that ethylene plays an essential role in dormancy induction.

## Ethylene as a Bloom-Delaying Agent

Ethylene has been used to delay bloom and avoid frost damage on fruit trees for decades. Ethephon is a plant growth regulator that degrades and releases ethylene once entering plant cytoplasm (Pahwa and Ghai, 2015). Many studies have reported that fall application of ethephon can effectively delay the blooming time in the following spring in many fruit species, especially stone fruits (**Table 1**). According to these studies, ethephon-induced bloom delay can range from 3 up to 18 days, depending on the concentrations and application time. In general, early application with higher concentration is more effective. For example, when applied at 10% leaf drop, 250 and 500 ppm ethephon delayed bloom in plums (Prunus domestica) by 13 and 16 days, respectively; whereas only 5 and 7 days bloom delay was observed when ethephon was applied at 50% leaf drop stage (Crisosto et al., 1990). The effectiveness of ethephon appears to be limited to the pre-dormancy stage, as serious flower bud abscission was induced when application was made after the satisfaction of CR (Durner and Gianfagna, 1991), and little or no effect was found when ethephon was applied in apricot during the dormancy stage (Grijalva-Contreras et al., 2011). In addition to delaying bloom date, fall application of ethephon was also found to enhance cold hardiness of dormant buds. In peach, a frost of -3.3°C during the bud expansion period resulted in a loss of 34% in the untreated buds, but only 8% mortality was recorded in the ethephon treated buds. Moreover, 30–40% of the buds treated with ethephon survived a -23°C mid-winter low temperature, whereas only 10% of untreated buds remained viable (Gianfagna et al., 1989). Similarly, Durner (1995) showed that ethephon treated peach flower buds were 0.8–2.8°C hardier than nontreated buds during mid-December through March in the northern East Coast. Practically, the consequences of delaying bloom and improving cold hardiness are significant, as lessening the effects of winter injury and spring frost damage can greatly contribute to the increase of crop yields.

**Table 1:** Effects of Autumn-Applied Ethephon on Fruit Trees.

Species	Conc. (mg L^-1^)	Bloom Delay by Days (days)	Yield Reduction	Injuries	Reference
Almond (*P. dulcis*)	75–300	7–9	Yes	No	(Grijalva-Contreras et al., 2011)
Apricot (*P. mume*)	100–300	3–7	NA	Lower fruit set, abnormal flowers, gummosis	(Moghadam and Mokhtarian, 2006)
Blueberry (*V. Cyanococcus*)	100–400	5–14	Yes	Late ripening	(Krewer et al., 2005)
Nectarine (*P. persica*)	50–400	6–15 (early) * 14–16 (late)	No (Year effect)	Leaf yellowing and defoliation	(Irving, 1987)
Peach (*P. persica*)	500 125–250	10–18 3–5	NA	Severe damage Light damage	(Coston et al., 1985)
Peach (*P. persica*)	60–120	NA	NA	Branch and trunk damage	(Funt and Ferree, 1986)
Peach (*P. persica*)	120	5–9	NA	NA	(Crisosto, 1989)
Peach (*P. persica*)	250–500	13–16 (10%) 5–7 (50%) **	Yes	Reduced flower density	(Crisosto et al., 1990)
Peach (*P. persica*)	100	3–10	No (Year effect)	Smaller fruit size, Late harvest	(Durner et al., 1990)
Peach (*P. persica*)	150	4.7	NA	Significant gummosis on scaffolds	(Deyton et al., 1992)
Peach (*P. persica*)	100–400	1–11	No (Year effect)	Floral bud death	(Sloan and Matta, 1996)
Peach (*P. persica*)	100–200	3–7	NA	NA	(Ebel et al., 1999)
Pistachio (*P. vera*)	250–750	5.4–12.6	No	No	(Askari et al., 2011)

Year effect, yield reduction was masked and confounded by the occurrence of spring frosts in the given year. NA, data not available. Asterisks (*, **), defoliation stages (time and %, respectively) when ethephon was applied.

## Limitations of Using Ethylene in Delaying Bloom

Despite the significant success in using ethephon to delay bloom and prevent spring frost, some studies have indicated beneficial effects of ethephon can be comprised by the occurrence of detrimental effects such as gummosis, leaf yellowing and abscission, terminal dieback, flower abscission, floral bud failure, low fruit set, and yield reduction (**Table 1**). Furthermore, fall application of 75–300 ppm ethephon on almond caused up to 3-fold yield reduction, and the yield reduction was in proportion to the ethephon concentrations (Grijalva-Contreras et al., 2011). Such linear yield decrease caused by increasing ethephon concentrations was also documented in peach (Crisosto et al., 1990). The formation and exudation of gums on the trunk or limbs of fruit trees, known as gummosis, is another major problem that pesters fruit growers when using ethephon. Tree gums are mainly composed of polysaccharides and are induced by various environmental stresses, mechanical or chemical injury, insect attack, or infection (Saniewski et al., 2006). Ethylene has been implicated as the leading factor that induces gummosis (Li et al., 2014). Ethylene can act synergistically with jasmonic acid to cause the breakdown of cell membranes and cell disintegration, which is the first step of the gum formation (Saniewski et al., 2006). Application of ethephon can induce gummosis in the bulbs of grape hyacinth (*Muscari armeniacum*) within several days (Miyamoto et al., 2010). Ethephon-induced gummosis in fruit trees, especially of stone fruit species, has been reported in many studies (Moghadam and Mokhtarian, 2006; Miyamoto et al., 2010; Miyamoto et al., 2019). Though literature lacks the data on the yield reduction due to ethephon induced gummosis, growth retardation and value loss caused by gummosis in fruit trees can be substantial (Beckman, 2003; Ezra et al., 2017). As ethephon-based PGRs are widely used in today’s fruit production practices, none of them are explicitly labeled as generic bloom-delaying agents for fruit trees in general. Recently, ethephon has been suggested to be potentially hepatotoxic (Bhadoria et al., 2018), and this may further limit the scope of its use.

## Possible Mechanisms by Which Ethylene Delays Bloom

Limited information is available on how the effect of a fall application of ethephon is carried over to influence the floral behavior during the following spring. The time of full bloom depends on the fulfillment of both chilling and HR; the former dictates endodormancy break and the latter, ecodormancy release (Campoy et al., 2012). Accordingly, the delayed bloom may result from an inadequate accumulation of chilling or heat. Indeed, previous studies have indicated that chilling accumulation negatively correlates with the number of days to full bloom and the HR (Durner and Gianfagna, 1991; Li et al., 2016). Durner and Gianfagna (1991) proposed that ethephon can reduce the effectiveness of chilling in breaking flower bud dormancy, as ethephon treated flower buds needed about 3 additional weeks of chilling exposure to reach the CR compared to controls. By examining excised peach branches, Coston et al. (1985) found that ethephon-treated flower buds grew slower than untreated buds in controlled conditions, even after the fulfillment of CR. Some early studies also supported this finding in which ethephon leads to altered bud responses to warm temperatures after endodormancy release, manifested as delay in the differentiation of flower buds and the growth rate (Crisosto, 1989; Gianfagna et al., 1989). These findings indicate that ethephon may prolong both endo- and eco-dormancy duration, and influence the springtime phenology of floral buds, which appear to develop slower responsiveness to seasonal changes. Particularly, if the ethephon increases the CR to the point that exceeds the local chilling hours, significant bloom delay will likely occur. Though many studies have gained some insights into the physiology of fruit trees influenced by ethephon, none of them have explored the underlying mechanisms of how ethylene affects bud ontogeny during endodormancy and their growth after dormancy break, especially at the molecular level, and more critical studies are still needed to answer these questions.

The Function of Ethylene in Dormancy Induction May Be Also Attributed to Its Interaction With Other Hormonal Pathways Such as ABA and GA, the Two Key Hormones That Act Antagonistically in Controlling the Establishment and Release of Bud Dormancy. It Has Been Observed That Ethylene Induces the Accumulation of ABA in Many Natural Physiological Processes (Grossmann and Hansen, 2001), Presumably Through Upregulating Genes Involved in the ABA Biosynthesis (Rodrigo et al., 2006). Indeed, the Ethylene Precursor, ACC, Was Found to Activate ABA Signaling Pathway in Leafy Spurge (*Euphorbia Esula*) (Dogramaci et al., 2013). the Critical Role of Ethylene in Dormancy Induction Has Become More Evident When an Ethylene-Insensitive Mutation (*Etr1*) in White Birch (*Betula Pendula*) Diminished ABA Accumulation and Delayed Dormancy Initiation (Ruonala et al., 2006). on the Other Hand, Studies in *Arabidopsis* Have Shown That Ethylene Signaling Components (I.E. CTR1, Erfs) Can Interfere With GA Signaling by Stabilizing DELLA Proteins, the Key Negative Regulator of GA Response, Leading to Growth Inhibition (Dubois et al., 2013; Dubois et al., 2015) and Bloom Delay (Achard et al., 2007). in Tobacco (*Nicotiana Tabacum*), Ethylene Was Also Found to Mediate in the Light (Low R:FR) Signal Transduction and GA Acts Downstream of Ethylene Signaling (Pierik et al., 2004). a More Detailed and Graphical Explanation of Ethylene Interactions With Other Pathways That Are Associated With Dormancy Induction Was Summarized in a Recent Review by Liu and Sherif (2019).

Considering the essential role of ethylene in plant defense and stress signaling pathways, the ethylene-induced bloom delay may be a result of the activated or amplified stress responses. If exogenous ethylene is sensed by plants as a stress signal, longer dormancy, and late flowering would be advantageous for perennial plants to survive the unfavorable conditions, since in terms of fitness, survival outweighs reproductive success in perennials (Shefferson, 2009). Some evidence has emerged to support this hypothesis. The aforementioned gummosis evidently links ethylene to plant defense network, as gummosis has a strict connection to defense response against biotic attacks (Saniewski et al., 2006). Moreover, a study in *Arabidopsis* showed that treatment of ethylene precursor ACC rapidly decreases cell proliferation rates by arresting the cell cycle, in a similar way as osmotic stress (Skirycz et al., 2011). Such ACC-induced arrest of cell cycle was shown to cause dwarfism in leafy spurge that mimics the dormant phenotype (Dogramaci et al., 2013). The high resemblance between stress and dormancy with regard to cell cycle arrest and growth inhibition suggests that they may share common or overlapping networks acting downstream of ethylene signaling pathway. Growth arrest proceeds, and is a prerequisite for the dormancy induction, and accelerated growth cessation has been suggested to be associated with greater dormancy depth and late bud burst (Kalcsits et al., 2009). In light of this, early growth arrest induced by exogenous ethylene may be responsible for the extended dormancy and bloom delay. Nevertheless, how precocious growth cessation intensifies dormancy and delays bud burst still needs further examination.

In a recent review, Beauvieux et al. (2018) highlighted the role of reactive oxygen species (ROS) as an important signal in mediating the progression of dormancy. The findings that ethylene enhances the production of ROS in response to stress (Zhang et al., 2016) and the ROS levels increase during the ethylene biosynthesis (Ionescu et al., 2017) point to the dual role of ethylene in: a) establishing basal level of ROS at the dormancy induction, and b) activating ROS detoxication mechanism to release dormancy by triggering transient ROS increase. Thus, if the fall-applied ethephon causes the ROS levels to be higher than what would be induced by natural environmental cues, such as short photoperiod (Karpinski et al., 2003) or cold temperature (Heidarvand and Amiri, 2010), intensified or extended dormancy will likely occur. It has been shown that stress can induce post-translational modifications. Although some modifications can be reset back to the basal level upon the relief of the stress, some can be carried forward as “stress memory” to exert long term effects (Chinnusamy and Zhu, 2009). Increasing evidence has indicated that histone acetylation is necessary for the transcriptional regulation of ethylene signal transduction elements, i.e. EIN3 and EIL1 (Wang et al., 2017; Zhang et al., 2017; Wang and Qiao, 2019). Therefore, if such ethylene mediated chromatin modifications are retained, they may likely affect the subsequent events of dormancy and flowering.

## Concluding Remarks and Future Perspectives

The phenology of plant dormancy and flowering are of great agricultural and economic importance. Ethylene exerts strong control over the progression of dormancy and flowering, and elucidation of its regulation mechanisms, especially its interaction and crosstalk with other signaling networks holds the key to formulate effective approaches for frost mitigation and avoidance. This review intended to summarize the current knowledge of ethephon-mediated bloom delay in temperate fruit trees and highlight the possible mechanisms by which ethylene interferes with the initiation, maintenance, and release of bud dormancy. As ethylene is at the hub of crossroads to defense- and stress-related signaling pathways, it might be meaningful to examine ethylene in the context of stress responses. Emerging evidence has suggested that pathways of stress response and dormancy may converge at the stages of growth cessation, and bud break. Nonetheless, specific questions that still need to be addressed may include: 1) how ethylene affects chilling and HRs in deciduous woody perennials; 2) how ethylene interacts with other regulatory networks such as those controlling flowering and cell cycle progression; 3) whether ethylene is modulating the level of DNA methylation and/or histone modification of dormancy-related genes, e.g. dormancy associated MADS (DAM) genes; and 4) how accelerated, rather than naturally induced, growth cessation is related to dormancy depth and budburst. Studies focusing on temporal transcriptomic and hormonal changes during the dormancy/growth cycle have indeed provided some valuable insights into the mechanisms by which ethylene mediates such multifaceted roles in bud dormancy, particularly in stone fruits. However, due to the wide range of climates under which these studies are conducted and to facilitate comparative analyses, it would be highly advisable that future investigations use chill units (CU) and growing degree hours (GDH), rather than Gregorian calendar, to time sample collections during the bud dormancy cycle.

## Author Contributions

JL and SS have contributed equally to the writing, editing, and preparation of this mini-review.

## Conflict of Interest

The authors declare that no conflicts of interest exist regarding the completion and publication of this research.

## References

[B1] Achard P. Baghour M. Chapple A. Hedden P. Van Der Straeten D. Genschik P. (2007). The plant stress hormone ethylene controls floral transition *via* DELLA-dependent regulation of floral meristem-identity genes. Proc. Natl. Acad. Sci. U.S.A. 104, 6484–6489. 10.1073/pnas.0610717104 17389366 PMC1851083

[B2] Askari E. Irani S. Razmjoo K. (2011). Bloom, maturity, and fruit set of pistachio in response to early season application of ethephon. Hortic. Environ. Biotechnol. 52, 29–34. 10.1007/s13580-011-0029-4

[B3] Augspurger C. K. (2013). Reconstructing patterns of temperature, phenology, and frost damage over 124 years: spring damage risk is increasing. Ecology 94, 41–50. 10.2307/23435667 23600239

[B4] Beauvieux R. Wenden B. Dirlewanger E. (2018). Bud dormancy in perennial fruit tree species: a pivotal role for oxidative cues. Front. Plant Sci. 9, 657. 10.3389/fpls.2018.00657 29868101 PMC5969045

[B5] Beckman T. G. (2003). Impact of fungal gummosis on peach trees. HortScience 38, 1141–1143. 10.21273/hortsci.38.6.1141

[B6] Bhadoria P. Nagar M. Bharihoke V. Bhadoria A. S. (2018). Ethephon, an organophosphorous, a fruit and vegetable ripener: has potential hepatotoxic effects? J. Family Med. Prim. Care 7, 179–183. 10.4103/jfmpc.jfmpc_422_16 29915756 PMC5958565

[B7] Bleecker A. B. Kende H. (2000). Ethylene: a gaseous signal molecule in plants. Annu. Rev. Cell Dev. Biol. 16, 1–18. 10.1146/annurev.cellbio.16.1.1 11031228

[B8] Campoy J. Ruiz D. Allderman L. Cook N. Egea J. (2012). The fulfilment of chilling requirements and the adaptation of apricot (Prunus armeniaca L.) in warm winter climates: An approach in Murcia (Spain) and the Western Cape (South Africa). Eur. J. Agron. 37, 43–55. 10.1016/j.eja.2011.10.004

[B9] Chinnusamy V. Zhu J. K. (2009). Epigenetic regulation of stress responses in plants. Curr. Opin. Plant Biol. 12, 133–139. 10.1016/j.pbi.2008.12.006 19179104 PMC3139470

[B10] Coston D. C. Krewer G. W. Elkner T. E. Williamson J. G. Sims E. T. J. (1985). Chemical treatments to delay bloom in peach prunus persica. J. Amer. Soc Hort. Sci. 110, 874–877.

[B11] Crisosto C. H. (1989). Fall ethephon delays -bloom in ‘redhaven’ peach by delaying flower differentiation and development during dormancy. J. Amer. Soc Hort. Sci. 114, 881–884.

[B12] Crisosto C. H. Miller A. N. Lombard P. B. Robbins S. (1990). Effect of fall ethephon applications on bloom delay, flowering, and fruiting of peach and prune. HortScience 25, 426–428. 10.21273/hortsci.25.4.426

[B13] Deyton D. Lockwood D. W. Ventataraman S. (1992). Effects of fall applications of ethephon and ga3 on peach trees. HortScience 27, 6. 10.21273/hortsci.27.6.690c

[B14] Dogramaci M. Foley M. E. Chao W. S. Christoffers M. J. Anderson J. V. (2013). Induction of endodormancy in crown buds of leafy spurge (*Euphorbia esula* L.) implicates a role for ethylene and cross-talk between photoperiod and temperature. Plant Mol. Biol. 81, 577–593. 10.1007/s11103-013-0026-3 23436173

[B15] Dubois M. Skirycz A. Claeys H. Maleux K. Dhondt S. De Bodt S. (2013). Ethylene Response Factor6 acts as a central regulator of leaf growth under water-limiting conditions in *Arabidopsis*. Plant Physiol. 162, 319–332. 10.1104/pp.113.216341 23553636 PMC3641212

[B16] Dubois M. Van Den Broeck L. Claeys H. Van Vlierberghe K. Matsui M. Inze D. (2015). The Ethylene Response Factors ERF6 and ERF11 antagonistically regulate mannitol-induced growth inhibition in *Arabidopsis*. Plant Physiol. 169, 166–179. 10.1104/pp.15.00335 25995327 PMC4577380

[B17] Durner E. F. (1995). Dormant pruning and fall ethephon application influence peach pistil hardiness. J. Amer. Soc Hort. Sci. 120, 823–829. 10.21273/jashs.120.5.823

[B18] Durner E. F. Gianfagna T. J. (1991). Ethephon prolongs dormancy and enhances supercooling in peach flower buds. J. Amer. Soc Hort. Sci. 116, 500–506. 10.21273/jashs.116.3.500

[B19] Durner E. F. Gianfagna T. J. Rooney F. X. Teiger G. S. Seiler M. J. Cantarella M. J. (1990). Harvest date and size distribution of peach fruit are altered with fall applied ethephon. HortScience 25, 911–913. 10.21273/hortsci.25.8.911

[B20] Ebel R. C. Caylor A. Pitts J. Boozer B. (1999). Effect of ethrel on bloom delay, harvest date, and fruit weight of `Empress’ peach. HortTech. 9, 65–67. 10.21273/horttech.9.1.65

[B21] Ezra D. Hershcovich M. Shtienberg D. (2017). Insights into the etiology of gummosis syndrome of deciduous fruit trees in Israel and its impact on tree productivity. Plant Dis. 101, 1354–1361. 10.1094/pdis-12-16-1836-re 30678598

[B22] Fan S. Bielenberg D. G. Zhebentyayeva T. N. Reighard G. L. Okie W. R. Holland D. (2010). Mapping Quantitative Trait Loci associated with chilling requirement, heat requirement and bloom date in peach (*Prunus persica*). New Phytol. 185, 917–930. 10.1111/j.1469-8137.2009.03119.x 20028471

[B23] Funt R. C. Ferree D. C. (1986). Ethephon induced bloom delay of peach trees, Ohio USA. Acta Hortic. 179, 163–170. 10.17660/ActaHortic.1986.179.14

[B24] Gianfagna T. Durner E. Teiger G. S. (1989). Reducing low temperature injury to peach flower buds with ethephon. Acta Hortic. 239, 203–206. 10.17660/ActaHortic.1989.239.27

[B25] Grijalva-Contreras L. R. Martínez-Díaz G. Macías-Duarte R. Robles-Contreras F. (2011). Effect of ethephon on almond bloom delay, yield, and nut quality under warm climate conditions in northwestern Mexico. Chil. J. Agr. Res. 71, 34–38. 10.4067/S0718-58392011000100004

[B26] Grossmann K. Hansen H. (2001). Ethylene-triggered abscisic acid: A principle in plant growth regulation? Physiol. Plant 113, 9–14. 10.1034/j.1399-3054.2001.1130102.x

[B27] Heidarvand L. Amiri M. (2010). What happens in plant molecular responses to cold stress? Acta Physiol. Plant 32, 419–431. 10.1007/s11738-009-0451-8

[B28] Hoffmann H. Rath T. (2013). Future bloom and blossom frost risk for *Malus domestica* considering climate model and impact model uncertainties. PloS One 8, 10. 10.1371/journal.pone.0075033 PMC3792985 24116022

[B29] Ionescu I. A. Lopez-Ortega G. Burow M. Bayo-Canha A. Junge A. Gericke O. (2017). Transcriptome and metabolite changes during hydrogen cyanamide-induced floral bud break in sweet cherry. Front. Plant Sci. 8, 1233. 10.3389/fpls.2017.01233 28769948 PMC5511853

[B30] Irving D. E. (1987). ‘Fantasia’ nectarine: Effects of autumn-appliedethephon on blossoming and cropping. New Zeal. J. Exp. Agr. 15, 67–72. 10.1080/03015521.1987.10425538

[B31] Kalcsits L. A. Silim S. Tanino K. (2009). Warm temperature accelerates short photoperiod-induced growth cessation and dormancy induction in hybrid poplar (Populus × spp.). Trees 23, 971–979. 10.1007/s00468-009-0339-7

[B32] Karpinski S. Gabrys H. Mateo A. Karpinska B. Mullineaux P. M. (2003). Light perception in plant disease defence signalling. Curr. Opin. Plant Biol. 6, 390–396. 10.1016/S1369-5266(03)00061-X 12873535

[B33] Krewer G. Nesmith D. S. Williamson J. Maus B. Mullinix B. (2005). Ethephon for bloom delay of rabbiteye and southern highbush blueberries. Small Fruits Rev. 4, 43–57. 10.1300/J301v04n01_06

[B34] Lang G. A. (1987). Dormancy: a new universal terminology. HortScience 22, 817–820.

[B35] Li Y. Fang W. Zhu G. Cao K. Chen C. Wang X. (2016). Accumulated chilling hours during endodormancy impact blooming and fruit shape development in peach (*Prunus persica* L.). J. Integr. Agric. 15, 1267–1274. 10.1016/S2095-3119(16)61374-6

[B36] Li Z. Zhu W. Fan Y. C. Ye J. L. Li G. H. (2014). Effects of pre- and post-treatment with ethephon on gum formation of peach gummosis caused by Lasiodiplodia theobromae. Plant Pathol. 63, 1306–1315. 10.1111/ppa.12214

[B37] Liu J. Sherif S. M. (2019). Hormonal orchestration of bud dormancy cycle in deciduous woody perennials. Front. Plant Sci. 10, 1136. 10.3389/fpls.2019.01136 31620159 PMC6759871

[B38] Liu Q. Piao S. Janssens I. A. Fu Y. Peng S. Lian X. (2018). Extension of the growing season increases vegetation exposure to frost. Nat. Commun. 9, 1. 10.1038/s41467-017-02690-y 29382833 PMC5789858

[B39] Ma Q. Huang J. G. Hanninen H. Berninger F. (2019). Divergent trends in the risk of spring frost damage to trees in europe with recent warming. Glob. Chang Biol. 25, 351–360. 10.1111/gcb.14479 30338890

[B40] Merchante C. Alonso J. M. Stepanova A. N. (2013). Ethylene signaling: simple ligand, complex regulation. Curr. Opin. Plant Biol. 16, 554–560. 10.1016/j.pbi.2013.08.001 24012247

[B41] Miyamoto K. Kotake T. Sasamoto M. Saniewski M. Ueda J. (2010). Gummosis in grape Hyacinth (*Muscari armeniacum*) bulbs: hormonal regulation and chemical composition of gums. J. Plant Res. 123, 363–370. 10.1007/s10265-009-0273-1 19941030

[B42] Miyamoto K. Saniewski M. Ueda J. (2019). Gummosis and leaf abscission in Yoshino cherry (*Prunus yedoensis*): relevance to hormonal regulation and chemical composition of gums. Acta Hortic. 1235, 467–474. 10.17660/actahortic.2019.1235.65

[B43] Moghadam E. G. Mokhtarian A. (2006). Delaying apricot (cv. Shahroudi) flower induction by growth regulators application. J. Appl. Sci. 6, 266–269. 10.3923/jas.2006.266.269

[B44] Nascimento F. X. Rossi M. J. Glick B. R. (2018). Ethylene and 1-Aminocyclopropane-1-Carboxylate (ACC) in plant-bacterial interactions. Front. Plant Sci. 9, 114. 10.3389/fpls.2018.00114 29520283 PMC5827301

[B45] Nzokou P. Paligwende N. (2008). The influence of three plant growth regulators on susceptibility to cold injury following warm winter spells in Fraser fir [Abies fraseri (Pursh) Poir] and Colorado blue spruce (*Picea pungens*). HortScience 43, 742–746. 10.21273/hortsci.43.3.742

[B46] Pahwa K. Ghai N. (2015). Effect of ethylene on physiological and biochemical parameters in different crop plants - A review. J. Nat. Appl. Sci. 7, 1064–1069. 10.31018/jans.v7i2.732

[B47] Pierik R. Cuppens M. L. Voesenek L. A. Visser E. J. (2004). Interactions between ethylene and gibberellins in phytochrome-mediated shade avoidance responses in tobacco. Plant Physiol. 136, 2928–2936. 10.1104/pp.104.045120 15448197 PMC523355

[B48] Rodrigo M.-J. Alquezar B. Zacarías L. (2006). Cloning and characterization of two 9-cis-epoxycarotenoid dioxygenase genes, differentially regulated during fruit maturation and under stress conditions, from orange (*Citrus sinensis* L. Osbeck). J. Exp. Bot. 57, 633–643. 10.1093/jxb/erj048 16396998

[B49] Rohde A. Bhalerao R. P. (2007). Plant dormancy in the perennial context. Trends Plant Sci. 12, 217–223. 10.1016/j.tplants.2007.03.012 17416545

[B50] Ruonala R. Rinne P. L. Baghour M. Moritz T. Tuominen H. Kangasjarvi J. (2006). Transitions in the functioning of the shoot apical meristem in birch (*Betula Pendula*) involve ethylene. Plant J. 46, 628–640. 10.1111/j.1365-313X.2006.02722.x 16640599

[B51] Ruttink T. Arend M. Morreel K. Storme V. Rombauts S. Fromm J. (2007). A molecular timetable for apical bud formation and dormancy induction in poplar. Plant Cell 19, 2370–2390. 10.1105/tpc.107.052811 17693531 PMC2002631

[B52] Saniewski M. Ueda J. Miyamoto K. Horbowicz M. Puchalski J. (2006). Hormonal control of gummosis in Rosaceae. J. Fruit Ornam. Plant Res. 14, 137–143.

[B53] Seeley S. D. Damavandy H. Anderson J. L. Renquist R. Callan N. W. (1992). Autumn-applied growth regulators influence leaf retention, bud hardiness, bud and flower size, and endodormancy in peach and cherry. J. Am. Soc Hortic. Sci. 117, 203–308. 10.21273/JASHS.117.2.203

[B54] Shefferson R. P. (2009). The evolutionary ecology of vegetative dormancy in mature herbaceous perennial plants. J. Ecol. 97, 1000–1009. 10.1111/j.1365-2745.2009.01525.x

[B55] Skirycz A. Claeys H. De Bodt S. Oikawa A. Shinoda S. Andriankaja M. (2011). Pause-and-Stop: The effects of osmotic stress on cell proliferation during early leaf development in *Arabidopsis* and a role for ethylene signaling in cell cycle arrest. Plant Cell 23, 1876–1888. 10.1105/tpc.111.084160 21558544 PMC3123952

[B56] Sloan R. C. J. Matta F. B. (1996). Peach bloom delay and tree responses to fall applications of ethephon. Miss. Agr. For. Expt. Sta. Bull. 1055, 9p.

[B57] Sumitomo K. Narumi T. Satoh S. Hisamatsu T. (2008). Involvement of the ethylene response pathway in dormancy induction in Chrysanthemum. J. Exp. Bot. 59, 4075–4082. 10.1093/jxb/ern247 18952907 PMC2639020

[B58] Suttle J. C. (1998). Involvement of ethylene in potato microtuber dormancy. Plant Physiol. 118, 843–848. 10.1104/pp.118.3.843 9808728 PMC34794

[B59] Tsipouridis C. Thomidis T. Xatzicharisis I. (2006). Effect of sprinkler irrigation system on air temperatures and use of chemicals to protect cherry and peach trees from early spring frost. Aust. J. Exp. Agr. 46, 5. 10.1071/EA04014

[B60] Unterberger C. Brunner L. Nabernegg S. Steininger K. W. Steiner A. K. Stabentheiner E. (2018). Spring frost risk for regional apple production under a warmer climate. PloS One 13, 7. 10.1371/journal.pone.0200201 PMC6059414 30044808

[B61] Vitasse Y. François C. Delpierre N. Dufrêne E. Kremer A. Chuine I. (2011). Assessing the effects of climate change on the phenology of european temperate trees. Agr. Forest Meterol. 151, 969–980. 10.1016/j.agrformet.2011.03.003

[B62] Vitasse Y. Schneider L. Rixen C. Christen D. Rebetez M. (2018). Increase in the risk of exposure of forest and fruit trees to spring frosts at higher elevations in switzerland over the last four decades. Agr. Forest Meterol. 248, 60–69. 10.1016/j.agrformet.2017.09.005

[B63] Wang K. L. Li H. Ecker J. R. (2002). Ethylene biosynthesis and signaling networks. Plant Cell 14 Suppl, S131–S151. 10.1105/tpc.001768 12045274 PMC151252

[B64] Wang L. Qiao H. (2019). New Insights in transcriptional regulation of the ethylene response in Arabidopsis. Front. Plant Sci. 10, 790. 10.3389/fpls.2019.00790 31275338 PMC6591485

[B65] Wang L. Zhang F. Rode S. Chin K. K. Ko E. E. Kim J. (2017). Ethylene induces combinatorial effects of Histone H3 acetylation in gene expression in *Arabidopsis*. BMC Genomics 18, 1. 10.1186/s12864-017-3929-6 28716006 PMC5512946

[B66] Zhang F. Wang L. Qi B. Zhao B. Ko E. E. Riggan N. D. (2017). EIN2 mediates direct regulation of histone acetylation in the ethylene response. Proc. Natl. Acad. Sci. U.S.A. 114, 10274–10279. 10.1073/pnas.1707937114 28874528 PMC5617289

[B67] Zhang M. Smith J. A. Harberd N. P. Jiang C. (2016). The regulatory roles of ethylene and Reactive Oxygen Species (ROS) in plant salt stress responses. Plant Mol. Biol. 91, 651–659. 10.1007/s11103-016-0488-1 27233644

